# Prediction of Physical Activity Patterns in Older Patients Rehabilitating After Hip Fracture Surgery: Exploratory Study

**DOI:** 10.2196/45307

**Published:** 2023-11-30

**Authors:** Dieuwke van Dartel, Ying Wang, Johannes H Hegeman, Miriam M R Vollenbroek-Hutten, Up&Go After a Hip Fracture Group

**Affiliations:** 1 Department of Biomedical Signals and Systems University of Twente Enschede Netherlands; 2 Department of Trauma Surgery Ziekenhuisgroep Twente Almelo Netherlands; 3 Ziekenhuisgroep Twente Academy Ziekenhuisgroep Twente Almelo Netherlands; 4 Board of Directors Medisch Spectrum Twente Enschede Netherlands; 5 See Acknowledgments

**Keywords:** continuous ambulatory monitoring, physical activity, pattern prediction, older patients, hip fracture rehabilitation, wearable sensing

## Abstract

**Background:**

Building up physical activity is a highly important aspect in an older patient’s rehabilitation process after hip fracture surgery. The patterns of physical activity during rehabilitation are associated with the duration of rehabilitation stay. Predicting physical activity patterns early in the rehabilitation phase can provide patients and health care professionals an early indication of the duration of rehabilitation stay as well as insight into the degree of patients’ recovery for timely adaptive interventions.

**Objective:**

This study aims to explore the early prediction of physical activity patterns in older patients rehabilitating after hip fracture surgery at a skilled nursing home.

**Methods:**

The physical activity of patients aged ≥70 years with surgically treated hip fracture was continuously monitored using an accelerometer during rehabilitation at a skilled nursing home. Physical activity patterns were described in our previous study, and the 2 most common patterns were used in this study for pattern prediction: the upward linear pattern (n=15) and the S-shape pattern (n=23). Features from the intensity of physical activity were calculated for time windows with different window sizes of the first 5, 6, 7, and 8 days to assess the early rehabilitation moment in which the patterns could be predicted most accurately. Those features were statistical features, amplitude features, and morphological features. Furthermore, the Barthel Index, Fracture Mobility Score, Functional Ambulation Categories, and the Montreal Cognitive Assessment score were used as clinical features. With the correlation-based feature selection method, relevant features were selected that were highly correlated with the physical activity patterns and uncorrelated with other features. Multiple classifiers were used: decision trees, discriminant analysis, logistic regression, support vector machines, nearest neighbors, and ensemble classifiers. The performance of the prediction models was assessed by calculating precision, recall, and *F*_1_-score (accuracy measure) for each individual physical activity pattern. Furthermore, the overall performance of the prediction model was calculated by calculating the *F*_1_-score for all physical activity patterns together.

**Results:**

The amplitude feature describing the overall intensity of physical activity on the first day of rehabilitation and the morphological features describing the shape of the patterns were selected as relevant features for all time windows. Relevant features extracted from the first 7 days with a cosine k-nearest neighbor model reached the highest overall prediction performance (micro *F*_1_-score=1) and a 100% correct classification of the 2 most common physical activity patterns.

**Conclusions:**

Continuous monitoring of the physical activity of older patients in the first week of hip fracture rehabilitation results in an early physical activity pattern prediction. In the future, continuous physical activity monitoring can offer the possibility to predict the duration of rehabilitation stay, assess the recovery progress during hip fracture rehabilitation, and benefit health care organizations, health care professionals, and patients themselves.

## Introduction

Physical activity is an important aspect in an older patient’s rehabilitation process after hip fracture surgery. Being physically active during rehabilitation results in faster improvement of mobility, faster independency in activities of daily living, and higher confidence in walking earlier in rehabilitation [1-5]. Continuous monitoring of physical activity during hip fracture rehabilitation can be used to assess how older patients progress in their physical activity over time [6,7]. Insights into an older patient’s physical activity progress is considered highly relevant, since this information can be used for a more proactive treatment policy, and it can provide personalized feedback on a patient’s physical activity level and recovery progress [8,9].

**Figure 1:**
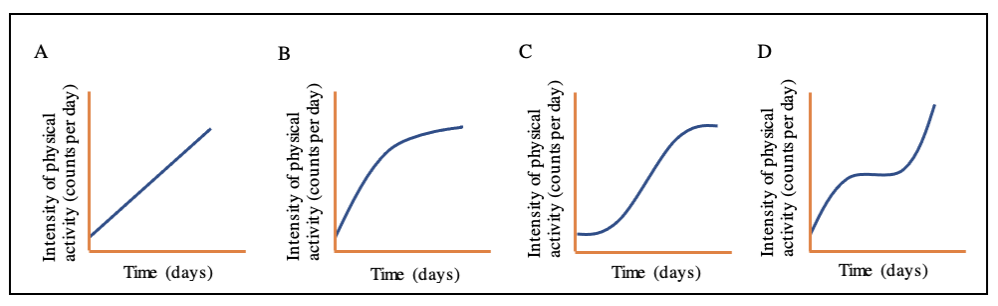
Patterns of overall physical activity in older patients rehabilitating at a skilled nursing home after hip fracture surgery [9]. (A) Upward linear pattern. (B) Hill-shape pattern. (C) S-shape pattern. (D) Cubic curve pattern.

Recently, we described the patterns and evolution of overall physical activity over time in older patients rehabilitating after hip fracture surgery [9]. Patterns were described for a sample of 66 older patients, and physical activity was continuously monitored during rehabilitation at a skilled nursing home by using a MOX wearable device, which was attached to the patients’ thigh. The results revealed different physical activity patterns when older patients were rehabilitating after hip fracture surgery. The most common pattern was the S-shape pattern (23/66, 35%), in which patients showed a slow increase in physical activity at the start of rehabilitation, followed by a steep increase and reaching a plateau at the end of rehabilitation. The other patterns found were the upward linear pattern (15/66, 23%), the hill-shape pattern (6/66, 9%), and the cubic curve pattern (6/66, 9%) (Figure 1) [9].

Knowing the expected physical activity pattern at an early stage of the rehabilitation phase could be clinically useful for multiple reasons. First, it could possibly contribute to an early indication of the duration of rehabilitation stay since our previous findings [9] showed a significant difference between the physical activity patterns and the duration of rehabilitation stay. Patients with the upward linear pattern had the shortest duration of rehabilitation stay (16 days) and patients with the cubic curve pattern the longest (42 days). Second, knowing the expected physical activity pattern at an early stage in the rehabilitation phase could provide health care professionals the ability to give patient-specific feedback and to assess a patient’s progress. Last, for patients, it could provide information about what to expect during rehabilitation. The next step to further investigate the patterns of overall physical activity over time is by exploring whether those patterns can be predicted early in the rehabilitation phase. There is no previous study that has predicted the recovery patterns of continuously monitored physical activity. Therefore, this study is an explorative study aiming to determine whether the recovery patterns of overall physical activity in older patients rehabilitating after hip fracture surgery at a skilled nursing home can be predicted at an early stage in the rehabilitation phase.

## Methods

### Study Design and Participants

This explorative study was conducted from January 2019 until June 2021 and was part of the “Up&Go after a hip fracture” project, which is a longitudinal observational study of older patients after hip fracture surgery with the aim to optimize the rehabilitation for these patients. The patient data of our previous study [9] about the patterns of overall physical activity were used for this study to explore whether the physical activity patterns can be predicted. A total of 66 older patients were enrolled in our previous study [9]. All patients were aged 70 years or older, surgically treated for a hip fracture at Ziekenhuisgroep Twente, and temporarily admitted to one of our collaborating skilled nursing homes (TriviumMeulenbeltZorg, Carintreggeland, or ZorgAccent) for geriatric rehabilitation. Exclusion criteria were severe cognitive impairment (ie, diagnosed with dementia), total hip replacement, pathological or periprosthetic fracture, plaster allergy, terminal illness, and contact isolation. Patients were enrolled 1 day before discharge to one of the collaborating skilled nursing homes.

### Ethics Approval

This study was approved by the Medical Research Ethics Committee Twente (K19-10) and by the institutional review board of Ziekenhuisgroep Twente (ZGT17-40), The Netherlands. The project was considered as not subject to the Medical Research Involving Human Subjects Act (Wet medisch-wetenschappelijk onderzoek met mensen). All patients gave written informed consent to participate. The privacy and confidentiality of our enrolled patients was achieved by storing the signed informed consent forms in a folder in a locked cabinet. Study data obtained from the enrolled patients were deidentified and stored at Castor electronic data capture or in a secure folder on the computer of Ziekenhuisgroep Twente.

### Study Procedure

All enrolled patients were continuously monitored during their rehabilitation at the skilled nursing home by using a MOX wearable device (Maastricht Instruments BV). The MOX is a small waterproof device, which consists of a triaxial accelerometer with a sample frequency of 25 Hz to measure physical activity from the lower extremities. The MOX was attached to the upper thigh of a patient, was capable of storing 1.5 GB of data, and had a battery life of 7 days. Raw MOX accelerometer data, measured during daytime (7 AM to 10 PM), were first preprocessed. A moving average filter with a window size of 0.12 seconds was used to eliminate noise acceleration [10]. A fourth order Butterworth High Pass filter was used with a cutoff frequency of 1 Hz to eliminate gravity acceleration [10]. Then, the overall intensity of physical activity measured at the lower extremities was calculated from the raw data as a parameter of physical activity by calculating the signal magnitude area, which is defined as the area under the curve of the accelerometer signals.

The overall intensity of physical activity was calculated per day and plotted for each rehabilitation day and each individual patient. First, to assess the physical activity patterns, figures were smoothed using a Gaussian-weighted moving average filter. Second, the physical activity patterns were visually analyzed by 2 experts in the geriatric rehabilitation field, who identified 6 unique physical activity patterns. Last, 18 independent raters visually analyzed and classified the physical activity pattern of each enrolled patient into one of the predefined unique patterns. When there were 2 patterns frequently chosen by the raters for a patient and the difference in the number of votes was equal to or smaller than 2, a final decision of the pattern was made by 2 experts in the geriatric rehabilitation field. Based on the visual analysis, 4 common patterns of overall physical activity were found in our previous study [9] for the 66 enrolled patients; a total of 15 (23%) patients were classified with the upward linear pattern, 6 (9%) patients with the hill-shape pattern, 23 (35%) with the S-shape pattern, and 6 (9%) with the cubic curve pattern. The remaining 16 patients (24%) were classified as “Else.” More details about the classification of the physical activity patterns are described in our previous paper [9].

For further analysis, we decided to focus on the prediction of the physical activity patterns of patients classified with the upward linear pattern (n=15) or the S-shape pattern (n=23), leaving the data of 38 patients for data analysis. This decision was made due to the small sample size of the patients classified with the hill-shape pattern and cubic curve pattern (n=6 for both patterns) and the high heterogeneity of the physical activity patterns in the “Else” group.

### Data Analysis

**Figure 2:**
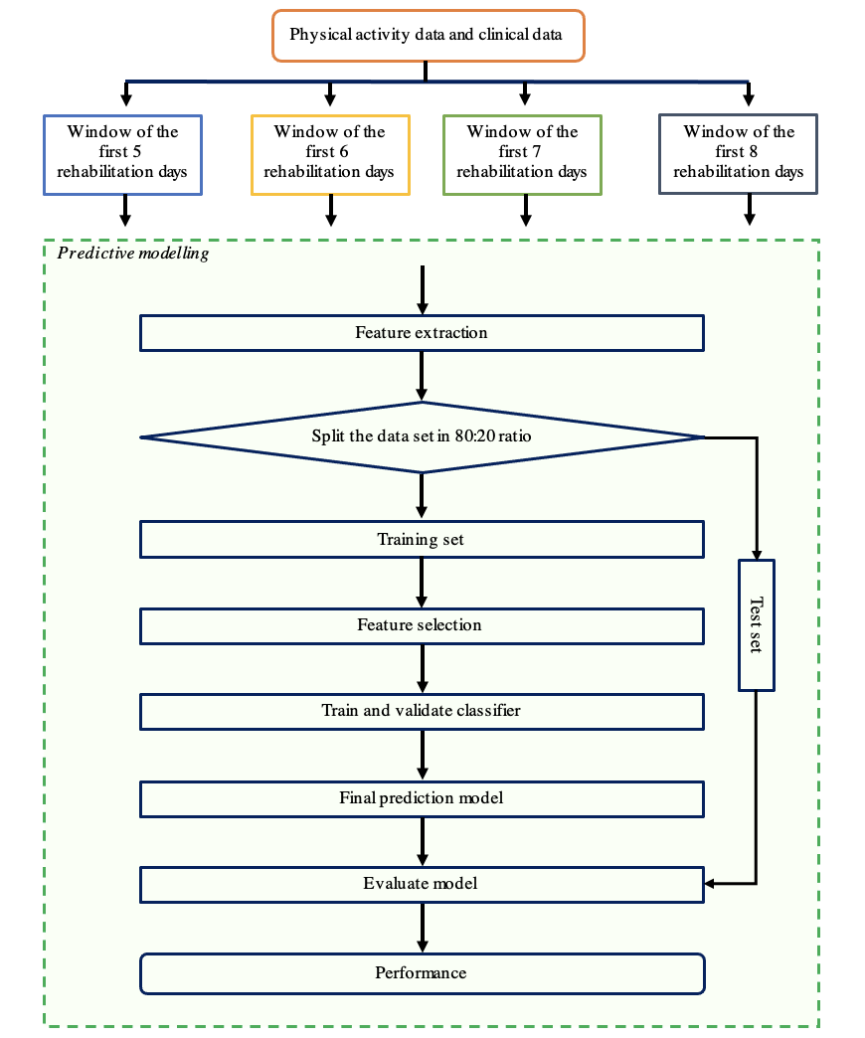
Workflow diagram for pattern prediction.

To predict the physical activity patterns, that is, the upward linear pattern or the S-shape pattern, we used the workflow diagram shown in Figure 2. First, features were extracted from the physical activity data and clinical data for 4 different time windows. These features were used as an input for the pattern prediction model. Second, patients were split into a training set and test set by using a ratio of 80:20, and relevant features necessary for the prediction model were selected. Third, the prediction model was trained and validated with the physical activity data of the patients within the training set, and lastly, the final prediction model was tested on the physical activity data of the patients within the test set.

#### Feature Extraction

Four types of features were extracted in this study: (1) statistical features, (2) amplitude features, (3) morphological features, and (4) clinical features. The statistical, amplitude, and morphological features were all extracted from the overall intensity of physical activity data. For the statistical features, the mean intensity, median intensity, SD, IQR, maximum intensity, minimum-maximum range, and root mean square values were calculated. For the amplitude features, the overall intensity at day 1 and the mean amplitude deviation were calculated. For the morphological features, the slope, characteristics of the third-degree polynomial curve describing the shape of the pattern, mean first order difference, and mean second order difference were calculated. The characteristics of the third-degree polynomial curve were defined as coefficient a, coefficient b, and coefficient c, which were derived from the third-degree polynomial equation: *y = ax^3^ + bx^2^ + cx + d*. More detailed information about the statistical, amplitude, and morphological features can be found in Multimedia Appendix 1.

To aim for early pattern prediction, we did not calculate the statistical, amplitude, and morphological features by using the overall intensity of physical activity data of the entire rehabilitation period. Instead, they were calculated for 4 different time windows containing the overall intensity of physical activity data of the early rehabilitation phase. The 4 defined time windows were a window of the first 5 rehabilitation days, a window of the first 6 rehabilitation days, a window of the first 7 rehabilitation days, and a window of the first 8 rehabilitation days. Multiple time windows were chosen to assess at which moment in the postoperative rehabilitation phase the physical activity patterns could be predicted most accurately. A window of 5 days was chosen as smallest window, since patients need time to get used to rehabilitation. The maximum window length of 8 days was chosen to aim for pattern prediction early in the postoperative rehabilitation phase. Statistical, amplitude, and morphological features were calculated for each defined time window.

Clinical features were extracted based on the results of our previous study [9]. The Barthel Index (BI), Fracture Mobility Score (FMS), Functional Ambulation Categories (FAC), and the Montreal Cognitive Assessment (MoCA) score were included, since all the features showed a significant or close to significant association with the physical activity patterns [9]. The BI described the level of activities of daily living independency at admission to rehabilitation and ranged from 0 (completely dependent) to 20 (completely independent) [11]. The FMS and FAC both scored the level of mobility at admission to the rehabilitation center. The FMS scored mobility regarding the use of walking aids and ranged from 0 (fully mobile without aids) to 5 (no functional mobility) [12]. The FAC scored mobility regarding the walking ability and ranged from 0 (not able to walk) to 5 (independent walking) [13]. The MoCA assessed the presence of mild cognitive impairment and ranged from 0 to 30 [14]. A score of 26 was considered normal.

All features were normalized between 0 and 1 by using min-max normalization because some features have a wide range of values, which can dominate over the features with a small range of values. Thus, through normalization, features with a wide range of values will not overrule the features with a small range of values, as shown as follows: x′ = (x – min(x)) / (max(x) – min(x)), where x is the original feature and x′ the normalized value of the feature.

#### Feature Selection

To evaluate the relevance of using each extracted feature and to prevent the prediction model from overfitting, the correlation-based feature selection (CFS) method [15,16] was used, which ranked all the features and selected a subset of relevant features based on the symmetrical uncertainty. A feature is defined as a relevant feature when it highly correlates with the physical activity patterns and is uncorrelated with the other features [15,16]. The symmetrical uncertainty is a normalized version of the information gain and ranges from 0 to 1, with 1 indicating a high correlation and 0 indicating no correlation [16]. Relevant features were selected when the symmetrical uncertainty between the feature itself and the class was higher than the predefined threshold and the symmetrical uncertainties between the feature and other features. The predefined threshold used for this study was 0.9. This threshold was chosen to only select features that were highly correlated with the class. Since the window size of the predefined time windows affected the values of the calculated features, feature selection was performed for each defined time window, resulting in a feature subset for each time window.

#### Training Prediction Model

The prediction of physical activity patterns, that is, the upward linear pattern and the S-shape pattern was performed by predictive modeling by using machine learning techniques. All patients were divided into a training set and a test set by randomly selecting 80% (30/37) of the patients for training and 20% (7/37) for testing. Division of the patients was performed proportionally to the physical activity patterns, where both the training set and test set contained roughly the same distribution of patients with the upward linear pattern and the S-shape pattern as in the total patient group of patients. For each time window, the selected features were entered into the classification learner app of MATLAB R2017b (MathWorks, Natick) to train and build an eventual prediction model. The physical activity patterns were predicted using classifiers, namely, decision trees, discriminant analysis, logistic regression, support vector machines, nearest neighbors, and ensemble classifiers. The trained classifiers were validated by the 5-fold cross-validation method. For each window, the classifier with the highest accuracy in the validation phase was chosen as the final prediction model for the test set.

#### Testing Prediction Model

The final prediction model obtained for the different time windows was tested on the patients within the test set. The overall performance of the prediction models was evaluated by calculating the precision, recall, and *F*_1_-score for each pattern of overall physical activity (ie, the upward linear pattern and the S-shape pattern) in each model. Precision characterizes the proportion of the correctly classified patients within a physical activity pattern (true positive) to the total number of patients classified with that pattern (true positive + false positive) and was calculated as follows: precision = true positive / (true positive + false positive) [17-19]. Recall characterizes the proportion of correctly classified patients within a specific physical activity pattern (true positive) to the total number of patients that actually have that specific pattern (true positive + false negative) and was calculated as follows: recall = true positive / (true positive + false negative) [17-19]. *F*_1_-score is a machine learning metric and measures the accuracy of the prediction model by combining the precision and the recall scores and ranges from 0 to 1 [19,20]. *F*_1_-score was calculated as follows: *F*_1_-score = (2 * true positive) / (2 * true positive + false positive + false negative). Since precision, recall, and *F*_1_-score were calculated for each individual physical activity pattern, an overall performance score was also calculated: the micro *F*_1_-score, which is the normal *F*_1_-score but then calculated for all N different physical activity patterns [20]. The micro *F*_1_-score is calculated as follows: micro *F*_1_-score = (2 * sum of the true positives for all patterns) / (2 * the sum of the true positives, true negatives, and false negatives for all patterns).

## Results

### Participant Data

A total of 38 out of 66 patients from our previous study [9] were included in this study. One patient was excluded from the analysis due to missing data in the first week of rehabilitation, leaving 37 patients for the analysis. Out of the 37 patients, 15 (41%) were classified with the upward linear pattern and 22 (59%) were classified with the S-shape pattern. The mean age of all the patients was 83.5 (SD 5.8) years, and 26 (70%) patients were females. A total of 36 (97%) patients lived at home before the hip fracture with or without help, and 1 (3%) patient lived in a residential home. The mean duration of the geriatric rehabilitation stay was 29 (SD 15) days.

### Pattern Prediction

#### Features

**Figure 3:**
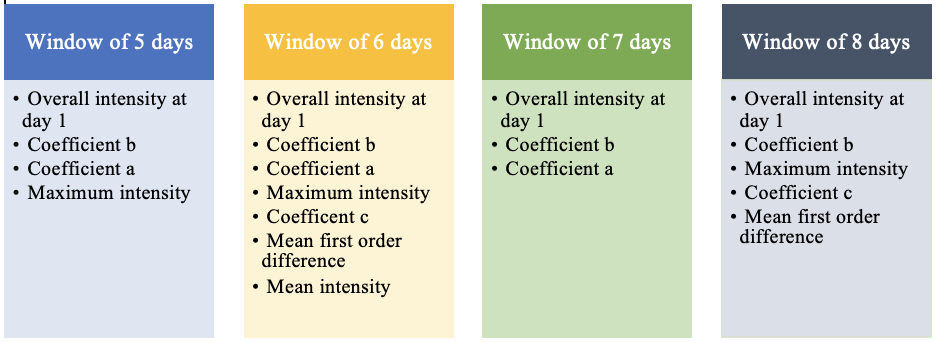
Feature subset for each time window selected by the CFS method.

Multiple features were selected by the CFS method for each time window (Figure 3). The overall intensity at day 1 and coefficient b were selected as relevant features for all time windows. Coefficient a was selected as a relevant feature in almost all time windows, except for the window of the first 8 days. The maximum intensity was selected as a relevant feature in almost all time windows, except for the window of the first 7 days. Coefficient c and the mean first order difference were only selected for the window of the first 6 days and the window of the first 8 days.

#### Prediction Model

A total of 30 patients were assigned to the training set and 7 patients were assigned to the test set. Table 1 presents the performance of the prediction model for each defined time window, where pattern 1 is defined as the upward linear pattern of overall physical activity and pattern 2 as the S-shape pattern of overall physical activity. The best performing prediction model was the model based on the time window of the first 7 rehabilitation days. For this time window, the final model was a cosine k-nearest neighbor prediction model. The results of using this prediction model on the test set showed the following performance: precision of 100%, recall of 100%, and an *F*_1_-score of 1 for predicting both the upward linear pattern of overall physical activity as well as the S-shape pattern of overall physical activity. The prediction model had an overall micro *F*_1_-score of 1.

**Table 1:** Pattern prediction performance of each time window^a^.

	Window of 5 days		Window of 6 days		Window of 7 days		Window of 8 days	
	Pattern 1	Pattern 2	Pattern 1	Pattern 2	Pattern 1	Pattern 2	Pattern 1	Pattern 2
Precision (%)	50	67	75	100	100	100	60	100
Recall (%)	67	50	100	75	100	100	100	50
*F*_1_-score	0.57	0.57	0.86	0.86	1	1	0.75	0.67
Final model	Cosine k-nearest neighbor		Linear support vector machine		Cosine k-nearest neighbor		Cosine k-nearest neighbor	

^a^Pattern 1 is defined as the upward linear pattern of overall physical activity. Pattern 2 is defined as the S-shape pattern of overall physical activity.

## Discussion

To the best of our knowledge, this is the first study to explore the ability of predicting recovery patterns at an early rehabilitation stage by using continuously monitored patterns of overall physical activity in older patients rehabilitating at a skilled nursing home after hip fracture surgery. The prediction of 2 different physical activity patterns was explored using the overall intensity of physical activity data in 4 different time windows. This resulted in 4 prediction models with a varying overall prediction performance. Using the window with overall intensity of physical activity data of the first 7 days of rehabilitation resulted in a prediction model with the highest overall prediction performance (micro *F*_1_-score=1). This was followed by the window of the first 6 rehabilitation days (micro *F*_1_-score=0.86), the window of the first 8 rehabilitation days (micro *F*_1_-score=0.71), and lastly by the window of the first 5 rehabilitation days (micro *F*_1_-score=0.57). Early prediction of the physical activity patterns in older patients during rehabilitation at a skilled nursing home after hip fracture surgery seems promising based on those first results, since these results indicate that we can distinguish between the 2 most common patterns, which gives the confidence to continue this research, explore the prediction of the other physical activity patterns, and further validate the results in a large number of patients.

As the results show, the prediction model using overall intensity of physical activity data of the first 7 rehabilitation days correctly predicted the physical activity patterns of all the patients within the test set (n=7). This suggests that at least 1 week of continuous physical activity monitoring is necessary to obtain the most accurate prediction of the physical activity patterns for the total rehabilitation period of older patients. Using only physical activity data of the first 7 rehabilitation days enables early prediction of the eventual physical activity patterns, which is considered highly relevant and clinically useful for multiple reasons. First, early prediction of the physical activity patterns can possibly give an early indication of the duration of rehabilitation stay at a skilled nursing home after hip fracture surgery, since our previous study showed a significant association between the patterns of overall physical activity and the duration of rehabilitation stay [9]. Unlike some other countries, the Dutch health care system does not have prefixed times for rehabilitation stay at a skilled nursing home after hip fracture surgery; therefore, it will benefit health care organizations if early prediction of the physical activity patterns can enable an early indication of the duration of rehabilitation stay. An early indication of the length of rehabilitation stay will not only optimize a patient’s discharge planning at the skilled nursing home but also give more insight into the availability of beds, which could optimize the patient flow between the hospital and the skilled nursing home. For health care organizations, this is beneficial since it can optimize capacity planning. For patients, a better transfer process from the hospital to the skilled nursing home could lower the duration of hospital stay, which results in a faster recovery process and a lower chance of in-hospital complications and mortality [21-24]. Second, early information about the expected pattern of overall physical activity can help patients manage their expectations of recovery. For patients, it is beneficial to know what to expect since it can prepare them for what is coming, manage their expectations, and help them set realistic goals. As shown by literature, patients need active involvement in their rehabilitation process to set realistic expectations and goals and to be more engaged during rehabilitation [25,26]. Therefore, it recommended to share the expected pattern of physical activity with patients and to actively involve them in their recovery progress, even if continuous physical activity monitoring shows a sudden deterioration in physical activity. Third, early information about the expected physical activity pattern can help health care professionals manage their expectations. This is beneficial for health care professionals since it enables them to continuously assess a patient’s progress as a result of the 24/7 continuous monitoring of physical activity. When the expected pattern of a patient is known, but if the pattern is suddenly different from expected, this is considered as being offtrack of the expected pattern. For example, when a patient’s physical activity level suddenly decreases when the expected physical activity pattern is an upward linear pattern, this may indicate sudden deterioration in the patient’s health condition due to some complication and clinicians should get some alarm bells for timely intervention. Early detection of a sudden deterioration in physical activity could encourage health care professionals to figure out what is causing this situation and intervene to prevent and reverse further deterioration. All of this promotes a patient-tailored rehabilitation program based on the needs and progress of each individual patient, which is also shown by literature as an important aspect for the recovery process of patients [25]. Last, early information about the expected physical activity pattern can motivate health care professionals to optimize the recovery process of older patients by trying to shift patients to a more favorable physical activity pattern. Furthermore, early information can also motivate patients to be more physically active. To decrease the duration of rehabilitation, for example, we can aim to shift patients to an upward linear pattern, when possible, since patients with this pattern had the shortest duration of rehabilitation stay. However, before this can be reached, research is first needed to assess which physical activity pattern is the most optimal pattern for older patients during hip fracture rehabilitation. Moreover, the exact activity levels within each pattern need to be assessed, and it needs to be assessed whether it is feasible to shift patients from one pattern to another. Additionally, it also recommended to perform more detailed research in the future about the association between the physical activity patterns and the duration of rehabilitation stay to assess whether there are some cofounding factors by using population-based statistical analysis to enrich knowledge for daily practice.

The intensity of overall physical activity on the first rehabilitation day was shown to be an important feature in predicting the physical activity patterns for all time windows. The intensity indicates that the level of physical activity at admission to rehabilitation is highly important in a patient’s subsequent physical activity pattern during rehabilitation. This result supports the importance of physical activity early in the postoperative rehabilitation phase and is in line with literature showing that higher physical activity levels result in a faster recovery in physical functioning [1,3,27]. This result can help health care professionals to understand the importance of early and frequent mobilization after hip fracture surgery. Furthermore, this finding suggests that health care professionals could focus even more on early mobilization during the in-hospital phase so that patients are discharged with higher physical activity levels to skilled nursing homes for rehabilitation.

Statistical, amplitude, and morphological features were extracted based on the intensity of physical activity data. Since this is the first study exploring the prediction of physical activity patterns, the statistical and amplitude features were chosen based on previous literature on machine learning algorithms for physical activity classification [28-31]. These features were common features and extracted from the time domain. Frequency domain features were not considered suitable for this study since the signals from the physical activity patterns were considered nonstationary. Additionally, morphological features were extracted from the overall intensity of physical activity data. The results of this study suggest that morphological features are also highly relevant in predicting the physical activity patterns for all time windows. Morphological features provided information about the shape of the patterns, which might explain their important role in this study, since the upward linear pattern and the S-shape pattern showed differences in their shape. The relevance of morphological features also stresses the importance of having continuously monitored physical activity data of older patients during hip fracture rehabilitation in contrast to using only the overall intensity of physical activity on the first day of rehabilitation. For future studies, it is recommended to keep focusing on the continuous monitoring of older patients with hip fracture and to focus on features related to the shape of the overall physical activity patterns.

In our previous study [9], patients with a higher BI score at admission to the rehabilitation center were more likely to be classified with the upward linear pattern of physical activity compared with patients with lower BI scores, which is in line with that reported in the literature [1,3,9,27]. However, clinimetric features such as the BI were not found in this study to be relevant for the prediction of the physical activity patterns. Pattern prediction was only predicted by features based on the continuously measured intensity of overall physical activity data, which is probably due to the higher range of those continuous physical activity features, since most clinimetric features were discrete variables. This result further supports the importance of continuously monitoring the physical activity of older patients during rehabilitation after hip fracture surgery.

Multiple classifiers were assessed in this study. The cosine k-nearest neighbor appeared to be the most accurate prediction model for all time windows, except for the window of the first 6 rehabilitation days. The principle underlying the k-nearest neighbor classifier is that it memorizes all training data, and based on those data, new unlabeled data can be classified. Features of new unlabeled data are compared against features in the complete training set, and the pattern of overall physical activity label of the k closest training data is used to determine the pattern of overall physical activity of the new unlabeled data [30,32,33]. However, there is no generally accepted classification method for predicting the physical activity patterns. The k-nearest neighbor is the first step to further explore the prediction of the physical activity patterns.

This study also had some limitations. The first limitation was the low number of enrolled patients, making this study an explorative study focusing on the prediction of only the 2 most common physical activity patterns. Although the results showed a 100% correct classification of the 2 most common physical activity patterns by using physical activity data of the first 7 days, the results need to be interpreted cautiously due to the low number of enrolled patients, and we cannot draw any firm conclusions yet. Therefore, it is recommended to continue investigating the patterns of overall physical activity in older patients rehabilitating after hip fracture surgery. For future studies, more patients should be included so that we can build stronger conclusions, assess more accurately which time window for feature calculation is the most optimal, include the prediction of the other physical activity patterns found in our previous paper (ie, hill-shape pattern, cubic curve pattern, and the “Else” group), and increase the generalizability [9]. Furthermore, to support the health care professionals, it is recommended to develop machine learning techniques on the common physical activity patterns found in our previous study [9], which can be used in future studies for automatic pattern recognition. In this way, visual analysis will be redundant. Additionally, for future research, it is recommended to use a Bluetooth version of the MOX accelerometer and to further develop and validate physical activity detection algorithms and prediction models, which can be integrated into a digital platform. By connecting the Bluetooth MOX accelerometer with the digital platform, live physical activity data can be obtained from a patient, which can be directly analyzed within the digital platform. In this way, using the MOX device is more applicable in clinical practice. A second limitation of this study is that we did not use more advanced techniques for feature selection and prediction modeling due to the explorative nature of this study. Even though the CFS considered the collinearity between features, it is recommended for future research to include a wrapper feature selection method, which is generally more accurate and tends to perform better than filter-based methods [34,35]. To train and validate the classifiers, we used MATLAB’s classification learner app. Using this learner resulted in highly valuable insights in the potential classifiers for pattern prediction. However, a disadvantage of using the classification learner is that there is less control of the hyperparameter optimization. For future studies, it is recommended to implement the hyperparameter optimization to improve the classification performance when using a short window size for feature extraction.

The aim of this explorative study was to investigate whether the 2 most common patterns of overall physical activity in older patients rehabilitating after hip fracture surgery can be predicted at an early stage of rehabilitation in the skilled nursing home. This study shows that the upward linear pattern and the S-shape pattern could be 100% predicted at an early stage of rehabilitation (within the first 7 days). The overall intensity of physical activity on the first rehabilitation day and morphological features were relevant features to predict those 2 common physical activity patterns. The results of this study seem promising for early prediction and can offer the possibility of predicting the duration of rehabilitation stay, assess the recovery progress during hip fracture rehabilitation, and benefit health care organizations, health care professionals, and patients themselves. More research is needed to further confirm the conclusions based on this study.

## Appendix

Multimedia Appendix 1 Detailed explanation of the extracted features.

## Abbreviations

BI: Barthel Index
CFS: correlation-based feature selection
FAC: Functional Ambulation Categories
FMS: Fracture Mobility Score
MoCA: Montreal Cognitive Assessment

## Data Availability

The data sets analyzed during this study are not publicly available since our patients did not give permission to share the data publicly. The data, however, are available from the corresponding author on reasonable request and with permission of the Up&Go after a hip fracture project. For data, please contact the corresponding author DvD (d.vdartel@zgt.nl).
