# Supplementary material for: Prediction of Physical Activity Patterns in Older Patients Rehabilitating After Hip Fracture Surgery: Exploratory Study
Source: JMIR Rehabil Assist Technol. 2023 Nov 30;10:e45307. doi: 10.2196/45307 (PMC10727481; doi:10.2196/45307)
Supplement: Multimedia Appendix 1 [file rehab_v10i1e45307_app1.docx]

Appendix 1. Detailed explanation of the extracted features

| **Feature** | **Explanation** |
| --- | --- |
| Mean intensity | The mean intensity was calculated to characterize the average value of the overall intensity of physical activity:  $Mean= \bar{x} = \frac{1}{n}\sum_{i=1}^{n} x_{i},$  where $x_{i}$ is the $i^{th}$ intensity value of overall physical activity in a window of $n$ data points. |
| Median intensity | The median intensity was calculated to characterize the “middle value” of the overall intensity of physical activity values in a dataset. |
| Standard deviation | The standard deviation was calculated to characterize the variability of the overall intensity of physical activity in a dataset and was defined as the square root of the variance:  $SD = \sqrt{\frac{\sum_{i=1}^{n} {(x}_{i}-{\bar{x})}^{2}}{n}},$  where $x_{i}$ is the $i^{th}$ intensity value of overall physical activity in a window of $n$ data points, and $\bar{x}$ the mean intensity of overall physical activity within that window. |
| Interquartile range | The IQR was calculated to characterize the statistical dispersion of the overall intensity of physical activity values in a dataset and was defined as the difference between the 75^th^ and the 25^th^ percentile of the overall intensity of physical activity values in a dataset. |
| Maximum intensity | The maximum intensity was calculated to characterize a patients’ maximum value of overall intensity of physical activity in a dataset. |
| Minimum-maximum range | The minimum-maximum range was calculated to characterize a patients’ range of overall intensity of physical activity within a dataset. |
| Root Mean Square | The RMS (equation 3) was used to characterize the quadratic mean of the overall intensity of physical activity and is defined as the square root of the mean square:  $RMS = \sqrt{\frac{x_{1}^{2}+ x_{2}^{2}+\ldots+ x_{n}^{2}}{n}},$ (3)  where $x$ is the overall intensity of physical activity and $n$ the number of data points within a window. |
| Overall intensity at day 1 | The overall intensity at day 1 was calculated to characterize the starting point of the intensity of physical activity at admission to the rehabilitation. |
| Mean Amplitude Deviation | The MAD (equation 4) was calculated to characterize the absolute variability of the overall intensity of physical activity and is defined as the average absolute difference between a set of overall intensity of physical activity values and the mean of these data points:  $MAD= \frac{1}{n} \sum_{i=1}^{n} {\vert x}_{i}-\bar{x}\vert, (4)$  where $x_{i}$ is the $i^{th}$ intensity value of overall physical activity in a window of $n$ data points, and $\bar{x}$ the mean intensity of overall physical activity within that window. |
| Slope | The slope was calculated to characterize the rate of change in the overall intensity of physical activity between the last and first overall intensity of physical activity value in a dataset. The slope was extracted by fitting the overall intensity of physical activity curve with a linear fit. Coefficient $a$ of the linear equation ($y=ax+b$) obtained from the linear fit was defined as the slope. |
| Coefficient a, b, c | Mathematical coefficients describing a third-degree polynomial curve (coefficient a, b, and c) were calculated to characterize the shape of the pattern of overall intensity of physical activity over time. All coefficients were extracted by fitting the overall intensity of physical activity curve with a third-degree polynomial curve fit. Coefficient $a$, $b$, and $c$ of the third-degree polynomial equation ($y={ax}^{3}+{bx}^{2}+cx+d$) obtained from the third-degree polynomial curve fit were defined as coefficient a, b, and c, respectively. |
| The mean first order difference | The mean first order difference and the mean second order difference were calculated to characterize the rate of change between data points of overall intensity of physical activity in a dataset. The mean first order difference was defined as the average of the differences between 2 consecutive values of overall intensity of physical activity within a dataset. |
| The mean second order difference | The mean second order difference was a follow-up from the first order difference. The second order difference was defined as the average of the differences between 2 consecutive differences found in the first order difference. |
